# An improved ChIP-seq peak detection system for simultaneously identifying post-translational modified transcription factors by combinatorial fusion, using SUMOylation as an example

**DOI:** 10.1186/1471-2164-15-S1-S1

**Published:** 2014-01-24

**Authors:** Chia-Yang Cheng, Chia-Han Chu, Hung-Wei Hsu, Fang-Rong Hsu, Chung Yi Tang, Wen-Ching Wang, Hsing-Jien Kung, Pei-Ching Chang

**Affiliations:** Department of Computer Science, National Tsing Hua University, Hsinchu, Republic of China: Taiwan; Biomedical Science & Engineering Center, National Tsing Hua University, Hsinchu, Republic of China: Taiwan; Department of Information Engineering and Computer Science, Feng Chia University, Taichung City, Republic of China: Taiwan; Department of Computer Science and Information Engineering, Providence University, Sha-Lu, Republic of China: Taiwan; Division of Molecular and Genomic Medicine, National Health Research Institutes, Miaoli County, Republic of China: Taiwan; UC Davis Cancer Center, Research III Room 2400, 4645 2nd Ave, Sacramento, CA 95817 USA; The institute for Translational Medicine, College of Medical Science and Technology, Taipei Medical University, 250 Wu-Xin Street, Taipei City, Republic of China: Taiwan; Institute of Microbiology and Immunology, National Yang-Ming University, Taipei, Republic of China: Taiwan

## Abstract

**Background:**

Post-translational modification (PTM) of transcriptional factors and chromatin remodelling proteins is recognized as a major mechanism by which transcriptional regulation occurs. Chromatin immunoprecipitation (ChIP) in combination with high-throughput sequencing (ChIP-seq) is being applied as a gold standard when studying the genome-wide binding sites of transcription factor (TFs). This has greatly improved our understanding of protein-DNA interactions on a genomic-wide scale. However, current ChIP-seq peak calling tools are not sufficiently sensitive and are unable to simultaneously identify post-translational modified TFs based on ChIP-seq analysis; this is largely due to the wide-spread presence of multiple modified TFs. Using SUMO-1 modification as an example; we describe here an improved approach that allows the simultaneous identification of the particular genomic binding regions of all TFs with SUMO-1 modification.

**Results:**

Traditional peak calling methods are inadequate when identifying multiple TF binding sites that involve long genomic regions and therefore we designed a ChIP-seq processing pipeline for the detection of peaks via a combinatorial fusion method. Then, we annotate the peaks with known transcription factor binding sites (TFBS) using the Transfac Matrix Database (v7.0), which predicts potential SUMOylated TFs. Next, the peak calling result was further analyzed based on the promoter proximity, TFBS annotation, a literature review, and was validated by ChIP-real-time quantitative PCR (qPCR) and ChIP-reChIP real-time qPCR. The results show clearly that SUMOylated TFs are able to be pinpointed using our pipeline.

**Conclusion:**

A methodology is presented that analyzes SUMO-1 ChIP-seq patterns and predicts related TFs. Our analysis uses three peak calling tools. The fusion of these different tools increases the precision of the peak calling results. TFBS annotation method is able to predict potential SUMOylated TFs. Here, we offer a new approach that enhances ChIP-seq data analysis and allows the identification of multiple SUMOylated TF binding sites simultaneously, which can then be utilized for other functional PTM binding site prediction in future.

## Introduction

SUMOylation was initially identified as a reversible post-translational modification that controls a variety of cellular processes including replication, chromosome segregation, and DNA repair [1–3]. The growing list of SUMO substrates includes various transcription factors (TFs) and chromatin remodeling molecules, which, upon SUMOylation, are often associated with transcriptional repression [4], and the maintenance of heterochromatin silencing [5, 6]. The deregulation of SUMOylation has been associated with a number of diseases including cancer [7–10]. SUMO has been found in all eukaryotes, but not in prokaryotes. Furthermore, the global regulatory role of SUMO in gene expression and protein interactions has been shown to be richly exploited in lower eukaryotes such as yeast [11, 12]. While numerous studies have provided considerable insight into the regulation of SUMOylated proteins in higher eukaryotes, their scope usually has been limited to a single host factor. The underlying complexity of SUMOylation has been extended by identifying the downstream consequences of these non-covalent interactions with effectors via SUMO interaction motifs (SIMs) [13], with the SIMs being critical to both SUMO conjugation and SUMO-mediated effects. Exploring the functions of SUMO conjugation and interaction during epigenetic regulation in mammalian cells will considerably enhance our knowledge of transcriptional regulation of SUMOylation in higher eukaryotes.

SUMOylation of transcriptional regulators results in alterations to the transcription regulation of individual genes, while the SUMOylation of epigenetic regulators brings about long-range chromatin remodeling, and hence global changes in expression. When chromatin structures are regulated by SUMO, it has been found to involve SUMO targeting of histone deacetylases and this then results in histone deacetylation, chromosome condensation, and transcriptional repression. At the same time, numerous transcription factors have been reported to be SUMO substrates, including Elk-1[14], SP1 [15], AP2[16], and many others. The study of epigenetic regulation when there is PTM of regulatory transcription factors is still in its infancy and there remains a need for new and improved screening tools as well as the development of assay pipelines.

Recently, chromatin immunoprecipitation (ChIP) followed by high-throughput sequencing (ChIP-seq), has become a powerful and high resolution method that allows the study of the impact of TFs and their co-regulators in higher eukaryotes in a genome-wide manner [17, 18]. During the ChIP process, DNA is initially cross-linked in a specific sample to the protein that binds to it. This cross-linked DNA is then broken into fragments and immunoprecipitation with a specific antibody for the DNA-binding protein follows; finally, the associated DNA is identified after de-crosslinking. High-throughput sequencing of short tags (reads) can be achieved using the resulting DNA population. ChIP-seq involves the short read (30~100 bp) sequencing of ChIP-enriched DNA fragments. These reads are subsequently aligned to a reference genome such as hg19. The first step of all ChIP-seq analyses is peak detection. Peaks are regions that are markedly enriched in terms of read density based on the ChIP-seq data. Potential transcription factor binding sites (TFBS) can only be precisely identified when the true peaks are detected first by "peak calling" tools.

**Figure 1:**
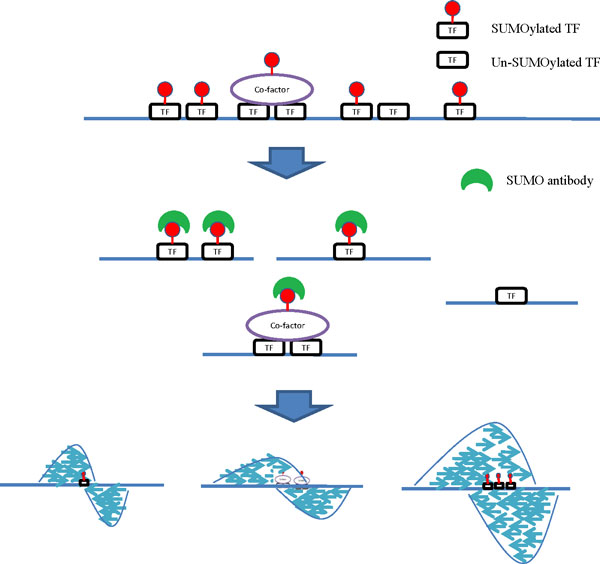
**Overview of experimental design**. The experimental design of the SUMO-1 ChIP-seq. DNA crosslinking with either SUMO-TF or SUMO-cofactor are identified using SUMO-1 antibody. Following size selection, all the resulting ChIP-DNA fragments were sequenced using an Illumina^®^ Genome Analyzer_*IIx*_.

Many peak calling algorithms have been developed for identifying ChIP-enriched regions from ChIP-seq experiments from a single TF [19]. In addition to commercial software, there are more than 30 open source programs available. Many reviews of the major steps in ChIP-seq analysis are available in literature [20–22]. These offer a variety of strategies that allow evaluation of each system and answer questions as to how to choose the most appropriate software from among the many available peak calling tools. Although current software is well established and can find the TFBS of single TFs, annotation of multiple functional TFBSs using the same PTM remains challenging [23]. TFs are known to recognize more than one motif and similar motifs can be recognized by different TFs. Simultaneously detecting the binding sites of multiple TFs, including SUMOylated TFs, is therefore a difficult task. Another big challenging is that the SUMO enriched sites represent not only directly SUMO modified TFs but also SUMOylated cofactors that are able to bind to the chromatin bound TFs (Figure 1). Therefore there is a wide range of discordance among the peaks identified by different software systems. This paper attempts to address the problem of predicting potential chromatin bound SUMOylated TFs and identifying their binding sites. To overcome the difficulty of simultaneously identifying SUMOylated TFs in ChIP-seq experiments, we investigated and compared the peak detection results of various different software approaches [24]. We selected four mainstream tools, Model-base Analysis of ChIP-seq (MACS) [25], T-PIC [26] , BayesPeak [27], and CisGenome [28]. MACS models uses the shift size of ChIP-seq tags to identify peaks and utilizes a dynamic Poisson distribution to highlight local biases in the genome. The "shift size" strategy of MACS helps to identify board and blunt peaks. However, this strategy may loss many sharp ones. T-PIC identifies significant peaks using topological data analysis and provides a non-parametric approach that is statistically sound and robust in relation to experimental noise. The T-PIC strategy is therefore able to identify most sharp peaks. Combine these two methods help us identifying most potential chromatin binding peaks. However, these two approaches may also identify some false positive peaks. The false positive peaks can be eliminated by combing peak detection methods, such as BayesPeak and CisGenome, that is specifically designed for identify the false positive peaks. BayesPeak was developed to model data structure using Bayesian statistical techniques. CisGenome was developed to model data structure using conditional binomial model. Combining BayesPeak or CisGenome with MACS and T-PIC using combinatorial fusion analysis [29], the results show that MACS*CisGenome*T-PIC (M*C*T) is superiors over MACS*T-PIC*BayesPeak (M*T*B). The M*C*T pipeline is able to improve peak detection in ChIP-seq data significantly. This approach should help produce great advances in our understanding of how SUMO modifications contribute to important biological processes.

## Results

### Global identification of SUMO-1 peaks in a primary effusion lymphoma (PEL) cell line, BCBL-1

**Figure 2:**
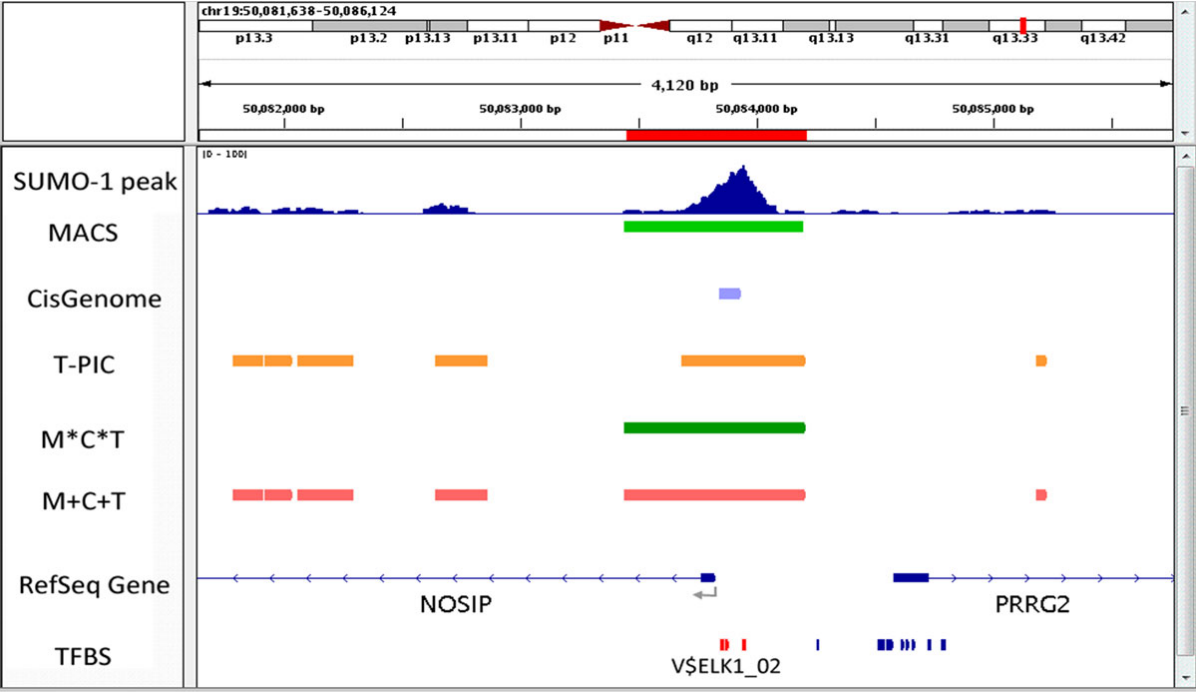
**Promoter region of NOSIP as explored by different peak detection methods with TFBS annotation**.

We used a ChIP-verified polyclone antibody specifically against SUMO-1 to immunoprecipitate SUMO-1 from a B lymphoma cell line, BCBL-1. Size-selected (400 bp) DNA fragments were excised and short reads (100 bp) obtained from both ends (paired-end reads) via high throughput sequencing-by-synthesis on an Illumina^®^ Genome Analyzer_*II*x _System. Analysing and interpreting ChIP-seq data typically involves pre-treating the raw reads using multiple applications, which can include mapping of sequences to the human genome, filtering and quality control. Around 63 million reads were mapped to the human genome sequence, hg19. Details of the number of reads that underwent data pre-process are presented in Table 1. After the density profiles were generated, the focus shifted to localizing the potential peaks. Here, we selected MACS, T-PIC, BayesPeak and CisGenome to localize the potential binding sites for delineated SUMO-1 targeting TFBSs. As shown in Table 2, the peaks calling results were found to be very different when the four different methods were compared. Specifically, MACS (M) detected 53,972 peaks with the longest regions (average 810 bp). T-PIC (T) detected the shortest peaks (average 442 bp). BayesPeak (B) and CisGenome (C) that were primarily designed to identify false positive peaks can be used to eliminate untrue peaks. Peaks sets identified by different methods were annotated using TFBSs (see materials and methods). T-PIC detected the greatest number of TFBS (477,353) in the whole genome, while MACS found the highest number of TFBS (27,615) in promoter regions. An example of the peaks identified by individual methods and their annotation by TFBS is presented in Figure 2. Consistent with other SUMO-1 ChIP-seq datasets (GEO ACCESSION: GSM1012775), we identified peaks in the promoter region of the NOSIP gene.

**Table 1:** SUMO-1 ChIP-seq alignment results

	SUMO-1 ChIP-seq data
**# of total reads**	97,620,354
**# of filtered reads**	70,300,792
**# of duplicate reads**	70,278,726
**# of mapped reads**	63,157,210
alignment rate*	89.87%

*The reads are aligned using BWA with the default parameters [39].

**Table 2:** Peak features obtained using the individual, union and intersection methods

	Peak #		TFBS peak #		TFBS #	
**Methods**	**Total**	**Promoter**	**Total**	**Promoter**	**Total**	**Promote**
MACS = M	53,972	10,282	15,428	3,934	110,779	27,615
CisGenome = C	32,158	12,069	7,153	4,640	30,828	5,322
T-PIC = T*	459,962	37,923	99,986	20,735	477,353	20,008
BayesPeak = B	241,257	35,349	102,905	9,188	220,182	48,710
M+C+T	465,103	31,096	100,484	15,352	460,753	39,449
M+T	460,503	38,023	100,417	20,775	477,996	22,042
M+C	65,605	32,588	17,643	7,400	120,964	54,569
C+T	462,622	43,355	100,284	20,735	478,565	22,008
M*C*T	20,349	9,834	4,834	3,604	20,525	3,860
M*T	50,655	17,246	15,274	9,312	128,473	10,526
M*C	20,525	9,852	4,863	3,612	20,643	3,874
C*T	30,158	11,914	6,780	4,637	29,616	4,956

* p-value < 0.001

### Intersection of different peak calling tools represents positive results

To evaluate the various individual systems and different combinations, we used four indexes: P_promoter_, P_TFBS_, P_tp_p_, and P_tp_t_ (see Methods section). The higher value of each index means that meaningful peaks were detected either in the promoter region or annotated TFBS. The results of the four individual tools are recorded in Tables 3. The order of average precision (AP) of individual tool is C (40.8%) > M (27.8%) > T (26.3%) > B ( 23.3%) (see Table 3). We choice the top three tools (C, M, and T) to do the following steps. All four combinations of intersection (*) and union (+) are recorded in Table 4 and 5, respectively. When we used the union and the intersection strategies to analysis the peaks of two or three tools, the average precision of intersection (M*C*T) was found to be the most effective method with highest average precision (45.8%) (Table 3 and 4). Three pools of SUMO-1 putative peaks in the promoter region were intersected to give 4,834 peaks. Among them, 3,604 peaks contain TFBSs. In total, 3,860 SUMO-1 related TFBSs were identified from these 3,604 peaks. This result indicates that the intersection method is able to extract functional peaks from a massive range of peaks.

**Table 3:** Precision indices for the single methods

Index	M	C	T	B
P_TFBS_	19.1%	37.5%	8.2%	14.7%
P_promoter_	28.6%	22.2%	21.7%	42.7%
P_tp_p_	38.3%	38.4%	54.7%	26.0%
P_tp_t_	25.5%	64.9%	20.7%	8.9%
AP	27.8%	40.8%	26.3%	23.3%

**Table 4:** Precision indices for the union (+) of two or three methods

Index	M+C+T	M+T	M+C	C+T
P_TFBS_	6.7%	8.3%	49.7%	9.4%
P_promoter_	21.6%	21.8%	26.9%	21.7%
P_tp_p_	49.4%	54.6%	22.7%	47.8%
P_tp_t_	15.3%	20.7%	41.9%	20.7%
AP	23.2%	26.3%	35.3%	24.9%

**Table 5:** Precision indices for the intersection (*) of two or three methods

Index	M*C*T	M*T	M*C	C*T
P_TFBS_	48.3%	34.0%	48.0%	39.5%
P_promoter_	23.8%	30.2%	23.7%	22.5%
P_tp_p_	36.6%	54.0%	36.7%	38.9%
P_tp_t_	74.6%	61.0%	74.3%	68.4%
AP	45.8%	44.8%	45.7%	42.3%

### Validation the data from ChIP-seq for ELK-1 binding sites with SUMO-1 enrichment by real-time qPCR

**Figure 3:**
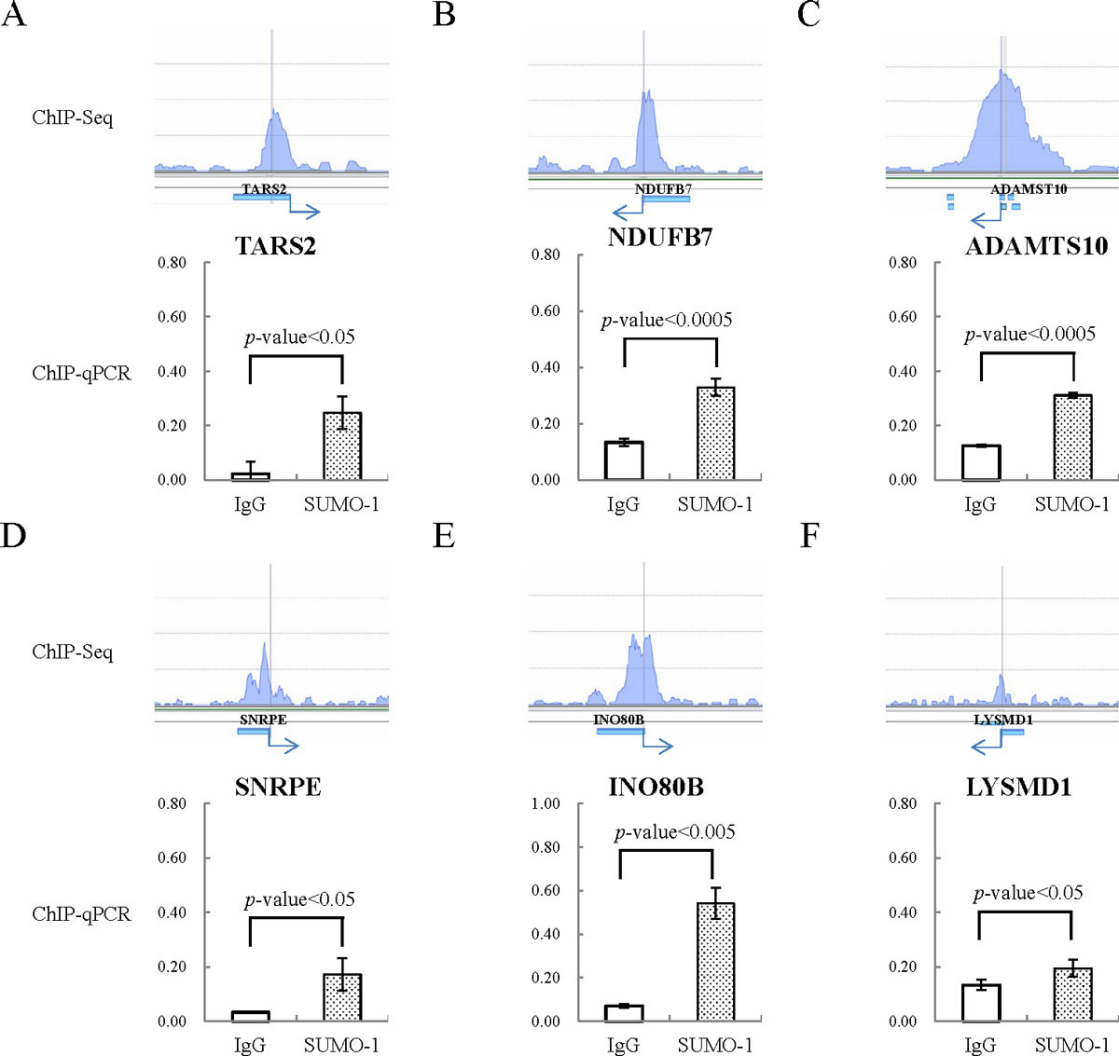
**Confirmation of ChIP-seq data for ELK1-binding sites with SUMO-1 enrichment in BCBL-1 cells using ChIP-qPCR**. Confirmation of data derived from ChIP-seq for ELK1 binding sites with SUMO-1 enrichment in BCBL-1 cells. The ELK1 binding sites within the SUMO-1 peak of the promoters of (A) TARS2, (B) NDUFB7 and (C) ADAMTS10 genes were amplified using qPCR. (D) SNRPE, (E) INO80B and (F) LYSMD1 genes identified in our SUMO-1 ChIP-seq result and GSM608163 ChIP-seq data were analyzed by qPCR with their specific primer pairs. All reactions were run in triplicate and normalized against the input. Nonspecific IgG was used as the control ChIP antibody.

**Figure 4:**
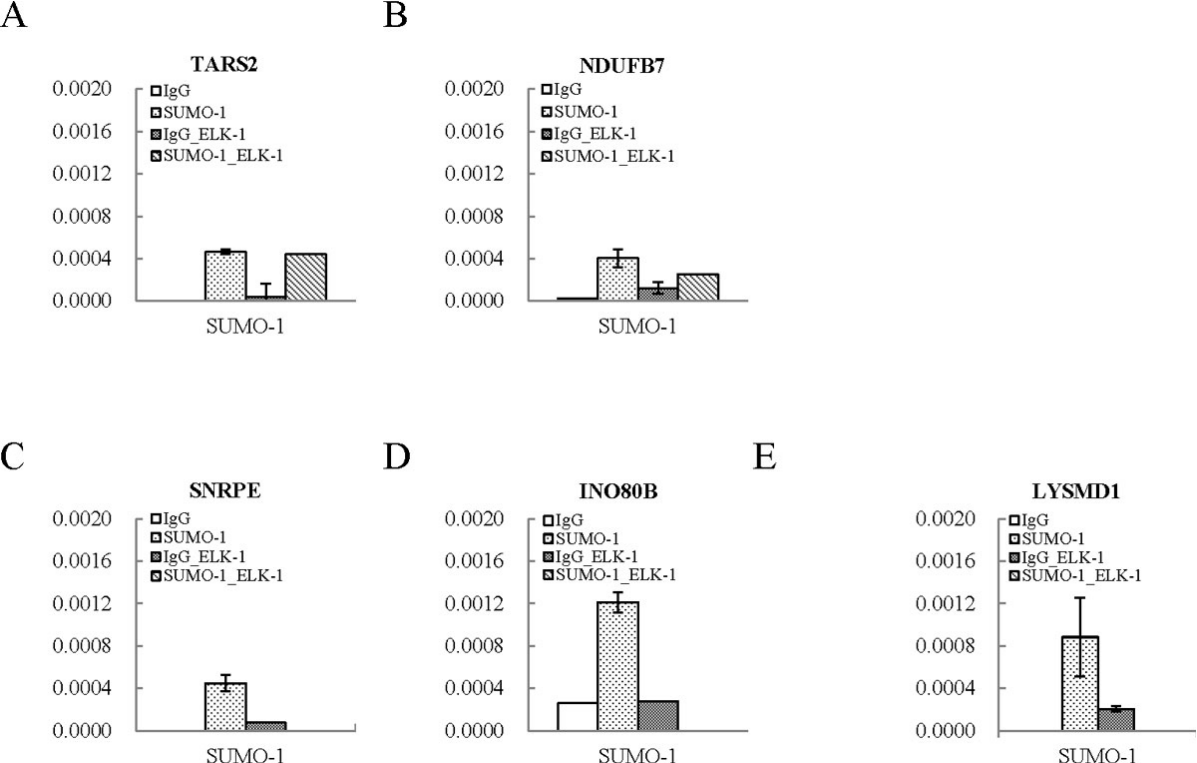
**Colocalization of ELK1 and SUMO-1 in the promoters of TARS2 and NDUFB7 genes**. Sequential chromatin immunoprecipitation (ChIP-reChIP) assay using control IgG and anti-SUMO-1 antibody for the first ChIP and anti-ELK-1 antibody for the reChIP was performed in formaldehyde-fixed chromatin derived fromTREx-F3H3-K-Rta BCBL-1 cells. Quantification of first ChIP and reChIP DNA recovered from (A) TARS2, (B) NDUFB7 and (C) SNRPE, (D) INO80B and (E) LYSMD1 by real-time qPCR using the promoter-specific primers.

To confirm the SUMO-1 enrichment at the ELK-1 binding sites, we randomly pick up three ELK-1 binding regions where the SUMO-1 peaks had been identified by the ChIP-seq assay and design primers for qPCR assay. The SUMO-1 enrichment in promoter regions of TARS2, NDUFB7 and ADAMTS10 was then validated using a ChIP sample and real-time qPCR. Consistent with the ChIP-seq results, the three ELK-1 binding regions tested here showed significant enrichment for SUMO-1 compares to IgG control (Figure 3A to 3C). ChIP-reChIP analysis was used to further confirm the co-localization of SUMO-1 and ELK-1 on ELK-1 binding sits of TARS2, NDUFB7 and ADAMTS10 promoter region with SUMO-1 enrichment. Control rabbit IgG and SUMO-1 antibody were used in the first ChIP, followed by reChIP using antibody for ELK-1. Real-time qPCR analyses of the first ChIP and reChIP product with TARS2 and NDUFB7 promoter-specific primers indicates that the SUMO-1 and ELK-1 are co-localized in the TARS2 and NDUFB7 promoter region (Figure 4A and 4B). Maybe due to the low PCR efficacy of ADAMTS10 promoter-specific primers, qPCR data show low detection value in the input of ADAMTS10 promoter region and no signal in ChIP and reChIP samples.

**Figure 5:**
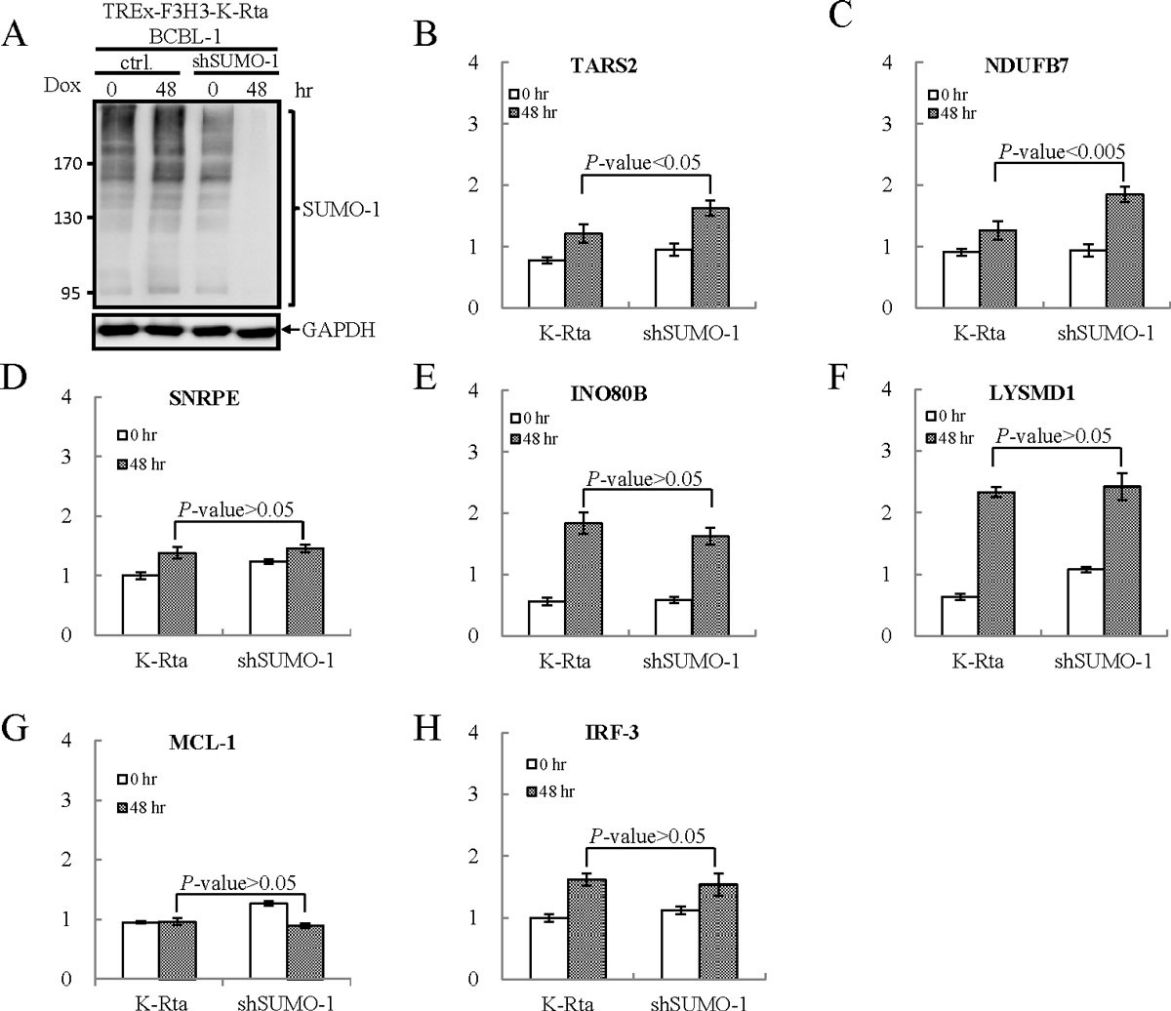
**Regulation of ELK-1 activity by SUMO-1 modification**. (A) TREx-F3H3-K-Rta-shSUMO-1 BCBL-1 cells were treated with Dox for 48 hours. TCLs were analyzed by immunoblotting using anti-SUMO-1 antibody. (B to H) Two ELK-1 targeted genes, TARS2 (B) and NDUFB7 (C), showing SUMO-1 enrichment at the promoter region identified in our study and three genes, SNRPE (D), INO80B (E) and LYSMD1 (F), that have high quality ELK-1 binding sites identified in HeLa cells overlapping with our SUMO-1 enriched regions were chosen. Two genes, MCL-1 (G) and IRF-3 (H), with ELK-1 binding site at the promoter region showing no SUMO-1 enrichment were chosen as control. RNA samples derived from TREx-F3H3-K-Rta BCBL-1 and TREx-F3H3-K-Rta shSUMO-1 BCBL-1 cells before and after 48 hours of Dox induction were subjected to reverse transcription (RT) reaction. Following the RT reaction, the ELK-1 target genes were amplified by qPCR using gene-specific primer sets. All reactions were run in triplicate and normalized against GAPDH.

To study the functional role of SUMO-1 in the regulation of ELK-1, we established a SUMO-1 inducible knockdown cell line in TREx-F3H3-K-Rta BCBL-1, namely TREx-F3H3-K-Rta shSUMO-1 BCBL-1. Effective knockdown of SUMO-1 protein in TREx-F3H3-K-Rta BCBL-1 cells was identified at 48 hours after Dox treatment (Figure 5A). Consistent with previous finding showing that SUMO modification of ELK-1 is required for its repressive activity [30], reverse transcription-qPCR (RT-qPCR) analysis showed a higher induction of TARS2 and NDUFB7 during K-Rta induced KSHV reactivation after SUMO-1 knockdown in TREx-F3H3-K-Rta shSUMO-1 BCBL-1 cells comparing with its parental TREx-F3H3-K-Rta BCBL-1 cells (Figure 5B and 5C).

### Potential SUMO-1 targeting TF identification that relies on SUMO-1ChIP peak height scores and can be validated via a literature review

**Figure 6:**
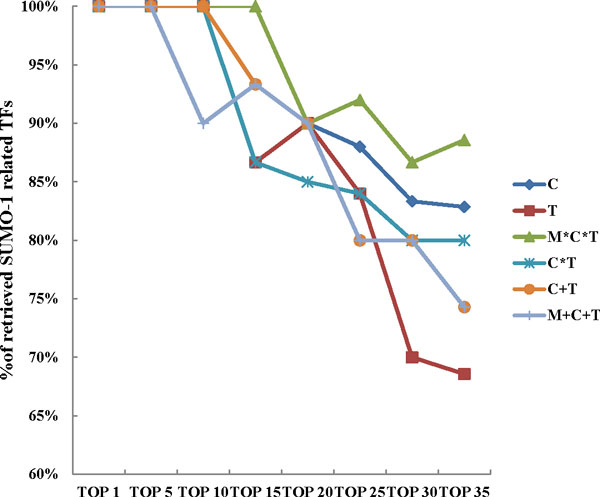
**Potential SUMO-1 TF verified result**. The percentage of literature verified SUMO-1 TFs predicted by the C, T, M*C*T, C*T, C+T and M+C+T methods, from top1 to top 35, plotted on a curve.

A score function, considering peak heights, frequency of TFBS on SUMO-1 peaks, and number of TFBS, was designed to predict SUMO-1 targeted TFs. Table 6 lists the 19 potential SUMO-1 targeting TF candidates predicted by the M*C*T method with Z-score using a cut-off value of 2.24. Literature-verified SUMOylation of the 19 potential SUMO-1 targeting TFs are presented in Table 6. The top five potential SUMO TFs, ELK-1 [30], E2F [31], NFY [32], and CREB [33], have all been confirmed to be SUMO substrates by literature review and the percentage of SUMO-verified TFs shown in Figure 6 indicates that the most favorable result is obtained when using the M*C*T combination.

**Table 6:** Potential SUMO-1 TF list

Rank	Transfac	Name of TF	SUMO related	Hampel identifier	Reference
1	V$ELK1_02	ELK1	Yes	9.88	[30]
2	V$E2F_02	E2F	Yes	7.01	[34]
3	V$E2F_03	E2F	Yes	5.97	[34]
4	V$NFY_01	NFY	Yes	5.90	[32]
5	V$CREB_Q2	CREB	Yes	5.55	[33]
6	V$CETS1P54_01	CETS1P54	Yes	4.14	[40]
7	V$NFY_Q6	NFY	Yes	3.93	[32]
8	V$SP1_01	SP1	Yes	3.89	[15]
9	V$STAT1_01	STAT1	Yes	3.72	[41]
10	V$AHRARNT_01	AHR	Yes	3.69	[42]
11	V$ATF_01	SP1	Yes	3.43	[42]
12	V$AHR_01	AHR	Yes	3.09	[42]
13	V$ELK1_01	ELK1	Yes	3.08	[30]
14	V$E2F_01	E2F	Yes	2.66	[34]
15	V$EGR1_01	EGR1	Yes	2.54	[43]
16	V$YY1_01	YY1	Yes	2.53	[44]
17	V$RFX1_02	hRFX1	Unknown	2.43	-
18	V$HEN1_02	NSCL1	Unknown	2.42	-
19	V$AP2_Q6	AP-2	Yes	2.40	[36]

Among the 19 potential SUMO TFs, 17 of them have been previously identified as SUMO substrates. For example, Elk-1, the top 1 SUMOylated TF candidate in our analysis, can be SUMO modified at its R motif [30]. Overall, 30% of the SUMO peaks (149/482) containing the Elk-1 TFBS that were identified in the present study are also found in another Elk-1 ChIP-seq data (GEO ACCESSION: GSM608163). Although no previous study reports have indicated that hRFX1 and NSCL1 are SUMOylated, we cannot rule out the potential of these two proteins to form a SUMO complex and/or to bind a SUMOylated cofactor.

### Validation of SUMO-1 enrichment in ELK-1 binding site identified in HeLa cells

Recently, a ChIP-seq report has pinpointed the global chromatin localization of ELK-1 in human HeLa cells (GEO ACCESSION: GSM608163). We selected three high quality ELK-1 binding sites identified in HeLa cells overlapping with our SUMO-1 enriched regions and validated by ChIP-qPCR. As shown in Figure 3D to 3F, there is a significant increase of SUMO-1 enrichment in ELK-1 binding sites of SNRPE, INO80B and LYSMD1 promoter identified from previous study of others in HeLa cells. However, ChIP-reChIP data shows no co-localization of ELK-1 and SUMO-1 in the promoter region of SNRPE, INO80B and LYSMD1 genes (Figure 4C to 4E). Consistent with the ChIP-reChIP data, the transcription of all three genes showed no changes during K-Rta induced KSHV reactivation after SUMO-1 knockdown comparing to the control cells (Figure 5D to 5F). The results were similar to the negative control genes, MCL-1 and IRF-3, which have ELK-1 binding site but not SUMO-1 enrichment in their promoter regions (Figure 5G and 5H). The inconsistency between our results and the findings in HeLa cells may be due to the cell type specificity. Together, taking ELK-1 as an example, the result here suggests that our pipeline is able to identify the potential chromatin region bound by SUMO modified transcription factors successfully. The biological role of SUMOylation in regulating the function of ELK-1 was also identified in a cell type-specific manner.

## Discussion

### Comparisons of the different methods available for the global identification of SUMO-1 peaks

**Figure 7:**
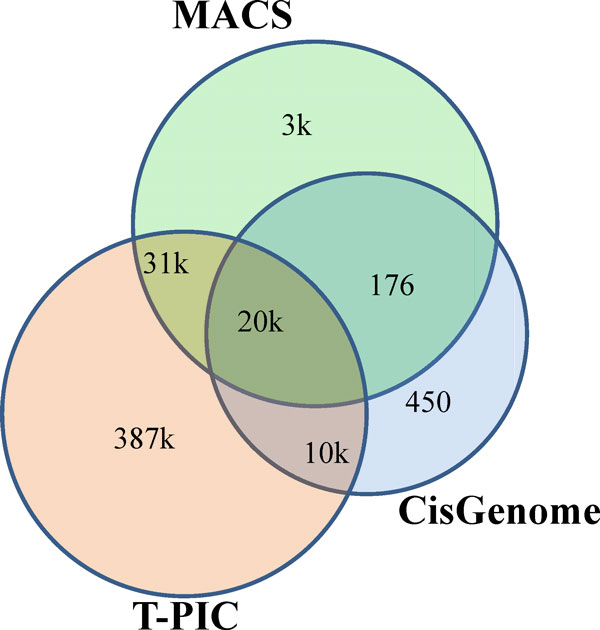
**Peak calling by different software**. The Venn diagram showing the overlaps among the peaks called by MACS, T-PIC and CisGenome, together with the numbers of peak presented. The numbers for the union and intersection of the peaks, and the mapped genes as obtained by the software can also be found in Table 4.

As revealed in Figure 7, different algorithms returned disjointed sets of peaks, which indicate that these divergent approaches and algorithms recognize distinct peaks. This finding creates a challenge as to how to integrate the results from different tools. Pepke et al. [20] classified the density profile of ChIP-seq result into three categories: (1) punctate regions; (2) broader regions; and (3) broad regions. Different strategies should be employed when delineating different profiles. Interestingly, evidence shows that SUMO-mediated transcription regulation not only involves covalent SUMO modifying transcription regulatory proteins, but also non-covalent associated co-regulatory proteins that contain the SUMO interacting motif (SIM). In most cases, SUMO formed complexes seems to result in regions that extend beyond a single TFBS, therefore rendering traditional peak calling methods inadequate when studying the binding sites for SUMOylation within long regions. An accurate characterization of the SUMOylation binding patterns will be of real significance to the study of SUMO binding sites across the entire genome, as well as any analysis of their correlation with transcriptional regulation.

### Evaluation of system fusion result

**Figure 8:**
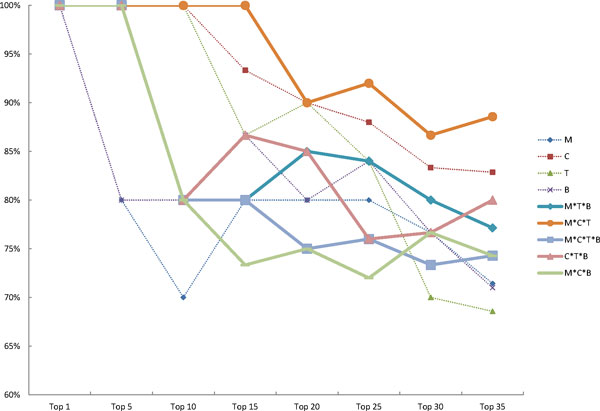
**Potential SUMO-1 TF verified result by the combinational methods of M, C, T and B**. The percentage of literature verified SUMO-1 TFs predicted by the combinational methods of M, C, T and B from top1 to top 35, plotted on a curve.

We performed two kinds of combination, intersection and union, with the four mainstream peak detection tools, namely MACS, T-PIC, BayesPeak and CisGenome (see Methods section). Intersection of two systems is expected to improve specificity, while union is expected to improve sensitivity. When evaluating each system or combination, we viewed the results with respect to combinatorial peaks using four percentage indices, P_promoter_, P_TFBS_, P_tp_p_, and P_tp_t_ (see Methods section). To evaluate these four indices, we created an average precision (AP). The results are shown in Tables 3 to 5. Table 3 lists the four indices from the four individual tools and each of thefour tools has its own strengths. MACS, T-PIC, BayesPeak and CisGenome detected the highest percentages of P_TFBS_, P_tp_p_, P_promoter_ and P_tp_t_, respectively. Table 4 showed that all combinations by union are negative cases with respect to the individual methods, due to an abundance of un-annotated peaks and intergenic peaks. As highlighted in Table 5, all combinations by intersection are positive cases, especially the M*C*T method. Collectively, each type of tool providing information beneficial to identify SUMO-1 peaks and the pipeline design here pinpoints potential SUMO-1 targeting TFs from others according to the scoring step. As shown in Figure 6, though the top 10 SUMO-1 targeting TF candidates are identified by M*C*T, the C+T, C*T, C and T methods provide similar SUMO verification rates. The verification rate for the following groups, namely top 15 to top 35, became lower compared to the M*C*T rate, C rate (the best individual method), C+T rate (the best union method) and M+C+T rate (the worst method of all). The results suggest that while all the methods are able to predict potential SUMO-1 targeted TFs when there is a strong peak score, the M*C*T method predicts SUMOylated TFs with a lower peak score in a more effective manner. In addition, we also compare the combination of all four methods of MACS, T-PIC, BayesPeak and CisGenome. As shown in Figure 8, combinational methods of M*C*T *B is not superior than M*C*T. The result indicates that the choice of peak calling tool is important. Using intersection strategy can filter the false positive peaks, however intersecting too many peak calling tools may let the unfit tool filter out the good peaks.

## Conclusions

Decoding how PTMs impacts the TF regulatory system that governs diverse cellular responses remains challenging. Taking SUMO modification as an example, we have developed a computational pipeline for predicting putative SUMOylated TFs from a group of TFBS. Using the combinatorial fusion methods described here, there is no need to depend on a single "best" algorithm. The merge detection method is able to find peaks with greater accuracy than any other peak calling software alone using ChIP-seq data retrieved from targeted PTMs. SUMO-1 target TFs are predicted well using our pipeline. In total, 89% of the 19 potential SUMOylated TFs were found to be SUMOylated after confirmation by literature review. In summary, our observations includes: (1) based on the criteria and performance evaluations used, there are no single answer to the selection of the best available method for ChIP-seq peak detection when identifying PTMs; (2) combinations of different tools are able to improve results in many cases; and (3) M*C*T is the superior prediction method when detecting putative SUMOylated TFs. More than 60% of the peaks identified in this study have not been annotated. One of the reasons for this is that the human cell contains thousands of TFs, and many of them are able to be SUMOylated. The TFBS data set from the UCSC genome browser only includes binding sites for 258 TFs out of these thousands of TFs. In the future, our work should help researchers to achieve a greater understanding of SUMOylated TFs once a better TFBS database become available. Moreover, we intend to explore the non-TFBS-annotated SUMO peaks in order to identify chromatin remodeling molecules that are not TFs. Most importantly, our pipeline here provides a platform for all researchers who want to study the relation between PTM and epigenetic regulation using their own chromatin binding data.

## Materials and methods

### Experiment design and sample preparation

The epigenetic study underling this paper's aim is an investigation of the impact of SUMO/TF interaction in a primary effusion lymphoma (PEL) cell line, BCBL-1. To this end, we generated ChIP-seq data using anti-SUMO-1 antibodies and BCBL-1. In general, the results of a SUMO-1 ChIP-seq experiment were anticipated to reflect indirectly the SUMO regulatory genome via SUMOylated TF binding and chromatin. In parallel to this, another scenario is that SUMO-1 antibody identifies SUMOylated cofactors that are recruited to TFs and TF-occupied DNA sequences. The cross-linked SUMO-TF-DNA complexes were extracted and contained the DNA that interacts with either the SUMOylated-TFs or the SUMOylated transcription complexes. After purification of ChIP-enriched DNA, a library was developed to allow sequencing on a NGS platform (Figure 1).

#### Cell culture

KSHV infected primary effusion lymphoma (PEL) cell line, TREx-F3H3-K-Rta BCBL-1 was grown in RPMI 1640 containing 15% FBS, 50 μg/ml blasticidin and 100 μg/ml hygromycin(Invitrogen, Carlsbad, CA) in the presence of 5% CO_2_.

The shRNA cassette of SUMO-1 (5'-CACCCAACACATCTCAAGAAACTCACGAATGAGTTTCTTGAGATGTGTTG-3') was inserted into pLenti4-T/O-shRNA plasmid and introduced into TREx-F3H3-K-Rta BCBL-1 cells by lentiviral transduction. Cells were selected for 14 days by 300 μg/ml zeocine (InvivoGen, ant-zn-1) and purified by Ficoll. Knockdown efficiency of SUMO-1 by shRNA were tested by treated the cells with doxycycline (Dox) for 48 hours. TREx-F3H3-K-Rta-shSUMO-1 BCBL-1 cells were maintained as described for TREx-F3H3-K-Rta BCBL-1 and supplemented with 300 μg/ml zeocine.

#### Chromatin immunoprecipitation-sequencing (ChIP-Seq), ChIP-reChIP assay and real-time quantitative PCR (qPCR)

1 × 10^7^ cells were harvested and ChIP assays were performed following the protocol described by the Farnham laboratory (provided at http://genomics.ucdavis.edu/farnham). ChIP-reChIP assays were performed by Re-ChIP-IT kit (Active Motif, Carlsbad, CA) following the manufacturer's instruction. ChIP-verified rabbit polyclone antibodies specific against SUMO-1 (Abcam, Cambridge, MA) and rabbit non-immune serum IgG (Alpha Diagnostic International) were used for the ChIP and ChIP-reChIP assays.

ChIP-seq library construction was carried out following the sample preparation protocol from Illumina. Short reads (100 bp) from both ends (paired-end sequencing) were sequenced on an Illumina^®^ Genome Analyzer_*II*X _System. The binding sites were verified by SYBR^®^ Green Based qPCR using a CFX connect™ real-time PCR detection system (Bio-Rad, Richmond, CA). Specific primer sets were designed around the identified binding sites for this purpose. Enrichment of SUMO-1 and IgG samples were normalized with the input.

### Data analysis

#### Input datasets

The reads within the SUMO-1 ChIP-seq data sets were aligned by BWA with default parameters [37]. hg19 was used as the reference genome, having been downloaded from the UCSC genome browser [38]. The Ensembl database was used to define gene regions [34]. Promoter regions are defined as the area that stretches from 5 kb upstream to 1kb downstream of a transcription start site (TSS).

#### Scoring system for TFBS in SUMO peaks

**Figure 9:**
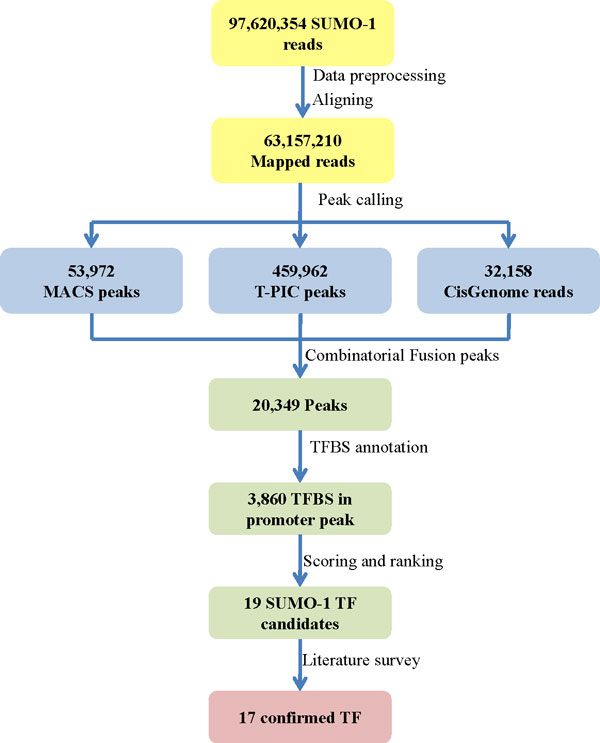
**Diagram of the SUMO-1 ChIP-seq analysis workflow**. Scheme used for the modified high-resolution ChIP-seq method and its validation. The literature was used to verify 17 of the top 19 SUMO-1-TF candidates. The SUMO-1-TF candidates were predicted by the following steps: (1) filtering poor and repeat reads out, and aligning reads to the human genome (hg19); (2) calling peaks using three tools MACS, T-PIC and CisGenome; (3) combining three peak sets; (4) annotating peaks using TFBS; (5) scoring and ranking SUMO-1 TF candidates; and finally (6) verifying SUMO-1 TF candidates via the literature.

Peak calling was the last, perhaps most pivotal and dynamic step in the process of the ChIP-seq pipeline after fragmentation, immunoprecipitation, sequencing and aligning. Figure 9 describes our pipeline for the SUMO-1 ChIP-seq experiment and the analytical workflow. The initial stage of peak detection was to identify the enriched regions with a large number of mapped reads. Subsequently, the peak calling tools had to determine if these regions were significant enough to represent a protein-DNA binding site across various peak heights and/or directionality score. This approach ensured that the peak heights are a scoring function in which the system assigned a number to each possible region. We propose that the peak detection for each of the binding sites be viewed as a scoring system on sets of all possible SUMO binding site regions, and the UCSC TFBS data set be viewed as known TFBS regions when annotating the SUMO binding site regions. The TFBS dataset was downloaded from the UCSC genome browser database, and includes a total of 5,797,266 TFBS for 258 TFs in Track TFBS [35].

Let *T* = [*t*_*1*_*, t*_*2*_*... t*_*258*_] be the set of TFs, and *TB*_*i*_, i = 1 ~258 be the set of TFBSs of *t*_*i*_. A range of SUMO peak detection scoring systems were developed using different algorithms. Using multiple scoring systems that were defined by the set of possible TFBS regions on the set of SUMO possible peaks, we were able to study the reproducibility of peak calls among different replicate. Multiple scoring systems were also used to develop and design new pipelines that had greater accuracy, efficiency and scalability when detecting SUMOylated protein binding sites during ChIP-seq data analysis. We drew from recent research on combinatorial fusion and applied this to ChIP-seq data analysis. Since peak heights were found to be the most consistent and best performing feature of peak calling methods, peak heights was selected as the score function to represent each method's scoring of the region identified. Let *D*_*x*_ be the set of peak regions identified by tool X, and *D*_*i*_^*x*^ be the intersection of *D*_*x*_ and *TB*_*i*_. The score function is defined as

It means the sum of the peak heights *D*_*i*_^*x*^ weighting with the percentage of *D*_*i*_^*x*^ in all *TB*_*i*_. Let the rank function R(*D*_*i*_^*x*^ ) be the function from 1 to 258 that is obtained by sorting the values in S(*D*_*i*_^*x*^ ) into descending order and converting the function S(*TB*_*x*_) into the function R(*TB*_*x*_) using the rank as its function value.

#### Combined two peak detection systems

##### Union

In the union of two systems, x and y, *D*_*x+y*_ is the set of regions that contains all peaks identified by X and all peaks identified by Y, where the overlapping regions between the two tools are merged to gather and form new compound regions, and non-overlapping peaks are allowed to maintain their genome position. Let *D*_*i*_^*x+y*^ be the intersection of *D*_*x+y*_ and *TB*_*i*_. The score function is

and R(*D*_*x+y*_) is the rank function obtained as R(*D*_*x+y*_).

##### Intersection

The intersection of two system, X and Y, *D*_x*y_ is the set of SUMO TFBS that are detected both by X and Y.

*D*_x*y_ ⊆ *D*_*x+y*_ where *D*_*x+y*_ = *D*_*x*_∩*D*_*y*_

The score function is

and R(*D*_x*y_) is the rank function obtained as R(*D*_x*y_).

#### Identifying potential SUMO-1 target TFs using the Hampel Identifier

Hampel identifier is a measure for the robustness of an estimator against outliers. It is regarded as one of the most robust and efficient outlier identifiers [36, 37]. The higher value of Hampel identifier means much more different from the main part of the data. We use Hampel Identifier [38] to identify the potential SUMO-1 targeting TFs. We apply Hampel Identifier on the score function S(*D*_*i*_^x^*^y^) which are estimates of and treat any observation as a potential SUMO-1 targeting TF for which the following is true:

where, M is the median of , ,... observations.

MADN = MAD/0.6745, and MAD is the median of the values ,  ,..., . 0.6745 is 0.75 quantile of standard normal distribution, and 2.24 is 0.975 quantile of chi-square distribution with one degree of freedom.

#### Performance evaluation methods

For many TFs, the majority of binding sites can be found near the TSS of expressed genes. Therefore, whether or not the peak is in the promoter region (promoter peak) can be an index when evaluating ChIP-seq software systems, and different combination methods. Thus, when, a peak overlaps with a TFBS, as a TFBS peak, this indicates that this is a functional peak. Thus, potentially, there is a percentage of TFBS peak found for all peaks and for promoter peaks, both of which represent evaluation indices. In this evaluation, we defined four indexes to compare the peaks identified by a particular tool and by combination of the three tools.

Meanwhile, average precision (AP) for a system is defined as

## Conflicts of interest

The authors declare that they have no competing interests.
